# Interleukin-4 induction of the CC chemokine TARC (CCL17) in murine macrophages is mediated by multiple STAT6 sites in the TARC gene promoter

**DOI:** 10.1186/1471-2199-7-45

**Published:** 2006-11-29

**Authors:** Kate Liddiard, John S Welch, Jean Lozach, Sven Heinz, Christopher K Glass, David R Greaves

**Affiliations:** 1 Sir William Dunn School of Pathology, University of Oxford, Oxford OX1 3RE, UK; 2 Department of Cellular and Molecular Medicine and Department of Medicine, UCSD, La Jolla, California 92093-0651, USA

## Abstract

**Background:**

Macrophages (Mθ) play a central role in the innate immune response and in the pathology of chronic inflammatory diseases. Macrophages treated with Th2-type cytokines such as Interleukin-4 (IL-4) and Interleukin-13 (IL-13) exhibit an altered phenotype and such alternatively activated macrophages are important in the pathology of diseases characterised by allergic inflammation including asthma and atopic dermatitis. The CC chemokine Thymus and Activation-Regulated Chemokine (TARC/CCL17) and its murine homologue (mTARC/ABCD-2) bind to the chemokine receptor CCR4, and direct T-cell and macrophage recruitment into areas of allergic inflammation. Delineating the molecular mechanisms responsible for the IL-4 induction of TARC expression will be important for a better understanding of the role of Th2 cytokines in allergic disease.

**Results:**

We demonstrate that mTARC mRNA and protein are potently induced by the Th2 cytokine, Interleukin-4 (IL-4), and inhibited by Interferon-γ (IFN-γ) in primary macrophages (Mθ). IL-4 induction of mTARC occurs in the presence of PI3 kinase pathway and translation inhibitors, but not in the absence of STAT6 transcription factor, suggesting a direct-acting STAT6-mediated pathway of mTARC transcriptional activation. We have functionally characterised eleven putative STAT6 sites identified in the mTARC proximal promoter and determined that five of these contribute to the IL-4 induction of mTARC. By *in vitro *binding assays and transient transfection of isolated sites into the RAW 264.7 Mθ cell-line, we demonstrate that these sites have widely different capacities for binding and activation by STAT6. Site-directed mutagenesis of these sites within the context of the mTARC proximal promoter revealed that the two most proximal sites, conserved between the human and mouse genes, are important mediators of the IL-4 response.

**Conclusion:**

The induction of mTARC by IL-4 results from cooperative interactions between STAT6 sites within the mTARC gene promoter. Significantly, we have shown that transfer of the nine most proximal mTARC STAT6 sites in their endogenous conformation confers potent (up to 130-fold) IL-4 inducibility on heterologous promoters. These promoter elements constitute important and sensitive IL-4-responsive transcriptional units that could be used to drive transgene expression in sites of Th2 inflammation *in vivo*.

## Background

Macrophages (Mθ) are key protagonists in both frontline defence against pathogens and regulation of the subsequent adaptive immune response. Chemokines secreted by Mθ during an inflammatory response are important determinants of the ensuing immune reaction, regulating the types and amounts of effector cells recruited to execute an appropriate response [1-3]. Alternatively-activated Mθ [4-6] play important roles in wound healing [7,8] and the resolution of inflammation [9,10], as well as contributing to allergic inflammation, hence IL-4-mediated signalling in Mθ is critical to many important human diseases.

The receptor for Thymus and Activation-Regulated Chemokine (TARC/CCL17), CCR4 [11], is most highly expressed on differentiated Th2 lymphocytes [12], as well as CD25^+ ^regulatory T cells [13] and CLA^+ ^(cutaneous lymphocyte antigen) skin-homing lymphocytes [14]. Thus, TARC is implicated in the recruitment of Th2 lymphocytes and the maintenance of Th2 immune responses [15], as well as in the suppression of classically-activated Mθ [16]. TARC expression levels correlate with the severity of disease in some chronic allergic pathologies, including asthma [17-19], atopic dermatitis [20] and cutaneous lupus erythematosus [21]. Additionally, *in vivo *neutralisation of TARC can limit Th2 lymphocyte recruitment and inflammation [22-24], underlining the significance of this chemokine to allergic pathologies and the importance of understanding how its expression is regulated in cells of the innate immune system.

Expression of the CC chemokine TARC has been shown to be up-regulated in human monocytes and Mθ treated with the Th2 cytokines, IL-4 and IL-13 [15,25], and inhibited by IFN-γ [25]. Murine TARC (mTARC[26]/ABCD-2[27]) shares 66% identity with human TARC at the amino acid level and is similarly chemotactic for CCR4^+ ^cells. mTARC has been shown to be expressed by Langerhans cells [28,29] and dendritic cells (DC) [30,31], but not by Mθ treated with IFN-γ and LPS [30]. The roles of alternatively activated Mθ in the production of mTARC and the mechanisms underlying the IL-4 induction of this chemokine in Mθ have not been fully addressed.

STAT6 is the sole STAT protein capable of signal transduction from the IL-4 receptor and activates the transcription of inflammatory mediators including eotaxin (CCL11) [32], Yml [33] and 12/15-lipoxygenase [34]. STAT6 also plays an integral role in the biology of alternatively-activated Mθ via activation of the myeloid transcription factor, Tfec [35] as well as arginase I [36]. Following ligand binding, JAK (Janus Kinase) proteins associated with the IL-4 receptor phosphorylate STAT6 monomers, allowing them to dimerise, translocate to the nucleus and activate gene transcription by binding to palindromic TTC(N)_4_GAA DNA consensus sites [37-39]. The C-terminus of STAT dimers contains the transactivation domain (TAD) [40], whereas the N-terminus may stabilise interactions between STAT dimers bound at tandem low-affinity sites less than 20 bp apart, resulting in enhanced transcriptional responses from these promoters [41].

In this paper, we demonstrate for the first time that IL-4 is a key inducer of TARC mRNA and protein in murine Mθ and confirm that this induction is mediated by the transcription factor, STAT6. Of eleven potential STAT6 sites identified within the murine TARC (mTARC) proximal promoter, we show that five of these sites contribute differentially and cooperatively to the IL-4-inducibility of the TARC promoter. These studies have also enabled us to generate a potent IL-4-inducible element based on the natural configuration of mTARC promoter STAT6 sites that could be used to drive expression of specific transgenes in response to IL-4 with potential therapeutic benefit.

## Results

### Murine TARC is up regulated by IL-4 in macrophages

**Figure 1:**
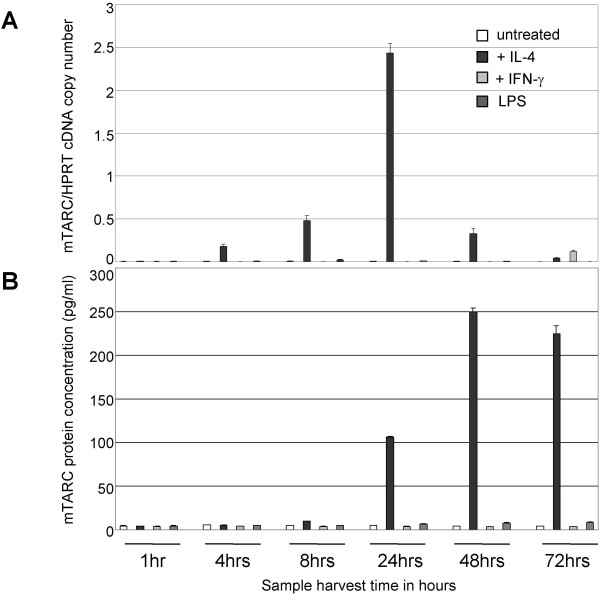
**mTARC mRNA and protein are strongly IL-4-induced and display temporal congruence**. C57BL/6 peritoneal BIOgel-elicited Mθ were cultured in OptiMEM alone (untreated) or in the presence of 20 ng/ml recombinant murine IL-4 or EFN-γ or 40 ng/ml LPS. *A*, Total RNA was harvested at the designated time points and subjected to real-time PCR. Relative mTARC cDNA copy number was interpolated from an internal standard curve and expressed as a ratio of mTARC/HPRT +/- SEM, the results shown are representative of more than 3 similar experiments. *B*, Supernatants were collected at same time points for protein determination by ELISA. Protein results represent data from duplicate samples +/- SD.

BIOgel-elicited peritoneal Mθ were stimulated with classical Thl^1 ^(IFN-γ) or Th2 (IL-4) cytokines or LPS (Figure 1). RNA and culture supernatant samples were simultaneously prepared from single wells at defined time points over a 72-hour period and were analysed for murine TARC (mTARC) mRNA and protein expression. Cell viability over this time period was independently confirmed by MTT assay (data not shown). mTARC mRNA was almost completely absent from untreated cells at all time points (Figure 1A), and was most potently induced by IL-4, consistent with its reported expression pattern in human Mθ [15,25]. Onset of mTARC induction by IL-4 was seen at 4 hours and peaked at 24 hours, when mTARC mRNA induction over untreated samples was more than 1,000-fold. IL-13 was also a potent inducer of mTARC mRNA at this time point, as measured by semi-quantitative PCR (data not shown). After 24 hours, there was a dramatic decline in mTARC mRNA levels detected in the IL-4-treated samples, although expression had still not returned to basal level within 72 hours. Neither LPS nor IFN-γ stimulated up-regulation of mTARC mRNA, even though these treatments resulted in significant induction of IP-10 (IFN-γ-inducible protein 10, CXCL10) [42] mRNA [see Additional file 1]. IFN-γ was also found to inhibit mTARC mRNA induction by IL-4 when added either prior to or concurrently with IL-4 treatment [see Additional file 1]. mTARC protein expression closely followed the mRNA profile in time of onset and duration (Figure 1B). As with the mRNA, mTARC protein expression was only detected in IL-4-treated samples. mTARC protein first appeared in the culture supernatants of IL-4-treated cells after 24 hours. Protein levels reached a peak at 48 hours, and had not declined greatly by 72 hours, as previously documented for human PBMCs [25] and T lymphocytes [43].

### Induction of TARC mRNA by IL-4 is mediated by STAT6 and does not require de novo protein synthesis

The kinetics of the IL-4 induction of TARC are such that several different mechanisms of promoter activation might be possible [44], including activation of PI3 kinase [45,46] or the IL-4-activated transcription factor, PPARγ [47,48]. However, mTARC induction by IL-4 was not inhibited by the PI3 kinase inhibitor, wortmannin, or the PKC inhibitor, chelerythrine chloride (data not shown) and mTARC mRNA was not up regulated by the PPARγ agonists, rosiglitazone, troglitazone or 15-deoxy-Δ^12,^^14^-prostaglandin J_2 _(data not shown). As STAT6-mediated gene transcription does not require *de novo *protein synthesis [49], we measured mTARC induction by IL-4 in the presence of the translational inhibitor, cycloheximide. Cells were pre-treated for 2 hours with doses of cycloheximide found to abrogate ^35^S-methionine incorporation. IL-4 was added and cells cultured for a further 4 or 24 hours before RNA harvest. mTARC mRNA was still induced by IL-4 in the presence of both cycloheximide doses at 4 hours and at 24 hours [see Additional file 2]. Furthermore, cycloheximide treatment resulted in mTARC superinduction, as has been noted for early immediate genes such as COX-2 [50]. These results indicate that *de novo *protein synthesis is not a pre-requisite of the IL-4 induction of mTARC and may actually act to reduce mTARC transcription.

**Figure 2:**
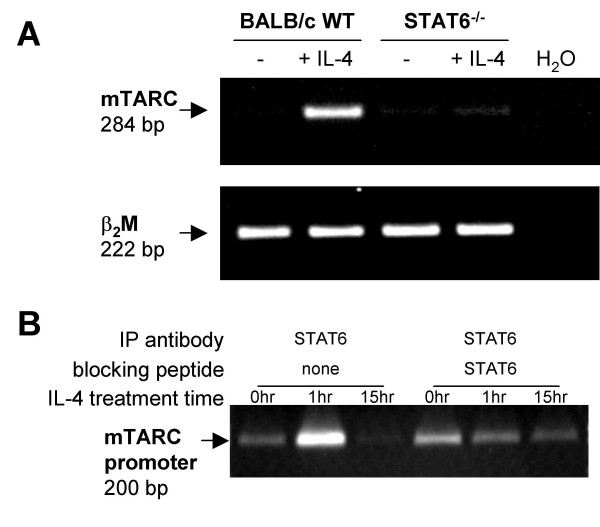
**The IL-4 induction of mTARC is STAT6-dependent**. *A*, Peritoneal thioglycollate-elicited Mθ from BALB/c wild-type or BALB/c STAT6^-/- ^mice were treated +/- lOng/ml recombinant murine IL-4 for 24 hours. Total RNA was harvested and subjected to semi-quantitative RT-PCR with mTARC or β_2_M (housekeeping gene) primers. **Chromatin immunoprecipitation experiments show STAT6 binding to the mTARC promoter**. *B*, Bone marrow-derived Mθ from Balb/c and STAT6^-/- ^were treated with 10 ng/ml recombinant murine IL-4 for the time periods shown before being formaldehyde fixed and sonicated. PCR using mTARC promoter primers was performed on DNA immunoprecipitated with STAT6 antibody in the presence or absence of a specific STAT6 blocking peptide. Amplification of 1/100^th ^of input DNA and no input DNA controls were routinely performed in each ChIP experiment for positive and negative controls, respectively.

To determine the involvement of STAT6 in mTARC regulation, thioglycollate-elicited peritoneal Mθ were isolated from both wild-type and STAT6^-/- ^[51] mice and treated with IL-4 for 24 hours (Figure 2A). Whereas the wild-type BALB/c Mθ exhibited the characteristic strong IL-4 induction of mTARC mRNA, the STAT6^-/- ^Mθ did not, demonstrating an absolute requirement of STAT6 for the IL-4-mediated up regulation of mTARC in Mθ. Binding of STAT6 to the endogenous mTARC promoter was also confirmed in ChIP assays using IL-4-treated bone marrow-derived Mθ (Figure 2B) in the presence or absence of a STAT6 blocking peptide. This experiment shows that STAT6 is rapidly recruited to the mTARC promoter in IL-4 treated Mθ.

### Identification of putative STAT6 binding sites in the murine TARC proximal promoter

**Figure 3:**
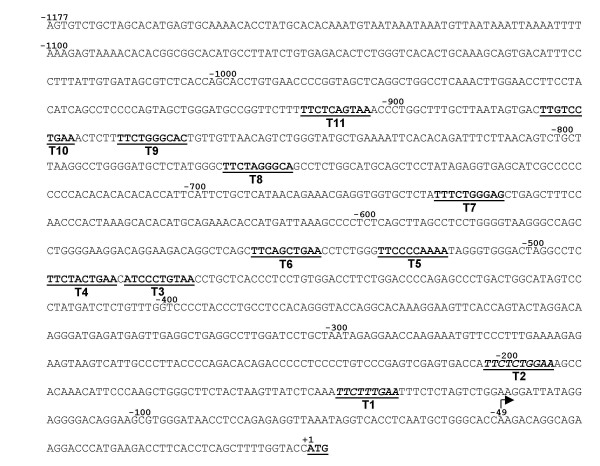
**-1173 bp mTARC promoter and putative STAT6 sites**. Sequence of the MDC-Fractalkine-TARC locus on mouse chromosome 8 was obtained from the Celera database and verified using the Sanger Centre database. Putative STAT6 sites in the proximal mTARC promoter were identified using low stringency searches using Genomatix MatInspector Release Professional 6.1 (November 2002) and the Transcription Element Search System (TESS). Sites conserved between human and mouse are written in italics. **ATG**indicates translation start codon, arrow indicates transcription start site and sequence positions are indicated as superscript, the start point of transcription of the mTARC gene as denoted + 1 [30].

We obtained the sequence for the MDC-Fractalkine-TARC loci on mouse chromosome 8 from the Celera and Sanger Centre [52] databases and identified eleven putative STAT6 binding sites (called STAT6 T1-T11 in order of proximodistal location) within a 1.2 kb region 5' of the mTARC coding sequence (Figure 3). Three of the putative mTARC STAT6 sites (T2, T4 and T6) conform to the published consensus sequence for STAT6 binding, TTC(N)_4_GAA [39]. The T1 site has TTC(N)_3_GAA spacing, to which STATs 1, 3, 4 and 5 [53-55] can bind, as well as STAT6 [33,56]. The remaining seven sites represent variations of the TTC(N)_4_GAA consensus, each possessing 4 central nucleotides flanked by the A and T residues that delineate the binding site. The T1 and T2 sites were found to be highly conserved between human and mouse, suggesting they may be of particular importance in mediating TARC induction by IL-4 in both species.

### Contribution of STAT6 sites T1-T11 to the IL-4 induction of murine TARC

**Figure 4:**
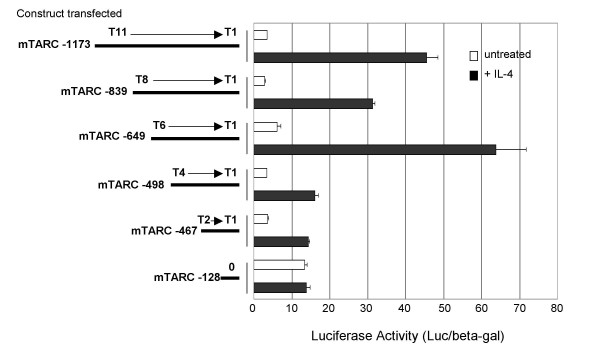
**The eleven putative STAT6 sites within the proximal mTARC promoter contribute differentially to the IL-4 induction**. Ten million RAW 264.7 cells were co-transfected with 5 μg mTARC promoter 5' deletion luciferase constructs and 3 μg STAT6 expression vector, β-galactosidase expression vector (1 μg) was also co-transfected to control for transfection efficiency. Identical transfections were pooled and plated into duplicate wells for each treatment. Cells were treated +/- 20 ng/ml recombinant murine IL-4 for 16–18 hours before being harvested and assayed for luciferase and β-galactosidase activity. Data are representative of 2 transfections and results are expressed as Luciferase Activity normalised for β-galactosidase activity +/- SD. Deletion construct end points are marked and correspond to the sequence information contained in Figure 3. The putative STAT6 sites contained in each construct are also indicated.

To analyse the relative contribution of sites T1-T11 to the IL-4 induction of transcription, we initially generated a 5' deletion series of promoter constructs in which these putative STAT6 sites were sequentially lost from a full-length -1173 construct containing all eleven putative sites and the transcriptional start site (Figure 4). The nomenclature of the mTARC promoter plasmids is derived from assigning the start point of transcription of the mTARC gene as + 1 [30]. These constructs were co-transfected into the RAW 264.7 Mθ cell line with a STAT6 expression vector to ensure transcription factor expression was not rate limiting or variable between samples. When stimulated with IL-4 for 18 hours, these deletion constructs demonstrated differential activation. Activation of full-length -1173 mTARC promoter was reproducibly induced at least 12-fold by IL-4, whereas the minimal mTARC promoter (mTARC -128), which lacks any STAT6 sites, was not at all IL-4-responsive. Deletion of DNA sequences between -649 and -498 (containing T5 and T6 sites) and -467 and -128 (containing T1 and T2 sites) reduces IL-4 induction, indicating that these two regions are important to the overall IL-4 response of the mTARC promoter.

**Figure 5:**
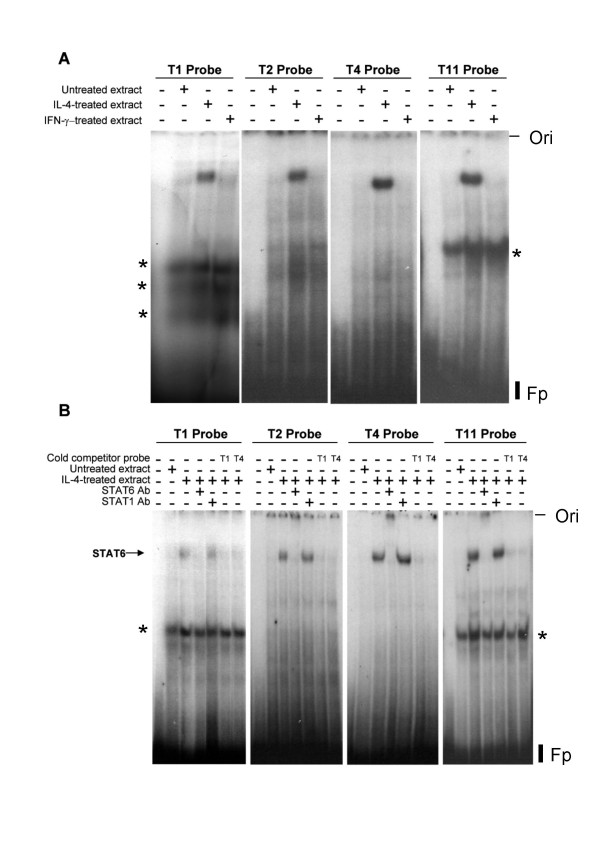
**Probes containing the mTARC putative STAT6 sites T1, T2, T4 and T11 bind stable STAT6 complexes *in vitro***. Nuclear extracts were prepared from C57BL/6 peritoneal BIOgel-elicited Mθ treated +/- 20 ng/ml recombinant murine IL-4 or TFN-γ for 1 hour. Extracts were incubated with radiolabelled mTARC T1-11 putative STAT6 site probes (data not shown for T3 and T5-T10, which did not bind STAT6) for 15 minutes before EMSA was performed (*A*). Specificity of binding was demonstrated by pre-incubating nuclear extracts +/- specific STAT Abs or excess cold competitor mTARC T1 or T4 putative STAT6 site probes (*B*). Bands labelled * represent nonspecific DNA-protein interactions, FP shows the top of the free probe and -Ori denotes the origin of the gel.

The STAT6 binding capacity of each putative STAT6 site was tested *in vitro *by EMSA (Figure 5). Nuclear extracts from C57BL/6 BIOgel-elicited Mθ untreated or treated with IL-4 or IFN-γ for 1 hour were prepared and incubated with radiolabelled probes for each individual mTARC STAT6 site. The probes containing the T2 and T4 STAT6 consensus sites were capable of binding a complex of the predicted mobility for STAT6 specifically present in extracts from IL-4-treated samples (Figure 5A). The T1 promiscuous STAT binding site and the T11 imperfect consensus site were also able to bind this complex. Surprisingly, the probe containing the T6 'perfect' STAT6 consensus site did not bind this complex (data not shown). The T3 and T5-T10 probes bound larger complexes that were not specific to IL-4-treated samples (data not shown). The IL-4-induced complex bound by T1, T2, T4 and T11 was supershifted by a STAT6-specific Ab, but not by a STAT1 Ab, further confirming that it contained STAT6 (Figure 5B). Both unlabelled T1 and T4 probes added in 100-fold molar excess were able to efficiently compete the STAT6 complex from the T1, T2, T4 and T11 probes. These experiments show that STAT6 can bind to sites with TTC(N)_3_GAA as well as TTC(N)_4_GAA spacing in the mTARC promoter and that perfect STAT6 consensus sequence is not the only determinant of STAT6 binding.

The binding capacity of an oligonucleotide *in vitro *is not always representative of its functional activity within cells [57] and so we compared the relative IL-4-inducibility of individual key mTARC putative STAT6 sites in transient transfection experiments. We generated luciferase reporter plasmids containing trimers of the T1, T2, T4 and T11 sites, which bound STAT6 in EMSA assays, and also of the T6 perfect consensus site, which did not bind STAT6, but has perfect consensus STAT6 binding sequence and might contribute to the activity of sequence region -649 to -498 (Figure 4).

**Figure 6:**
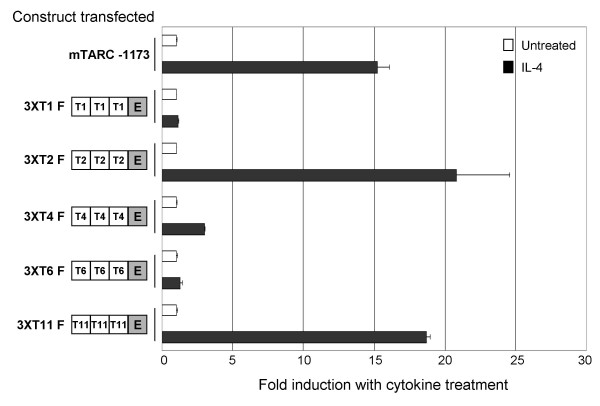
**Constructs containing trimers of mTARC STAT6-binding sites are induced to varying degrees by IL-4**. RAW 267.4 cells were transfected as described in Figure 4A with 5 μg luciferase-conjugated mTARC STAT6 site trimers and 3 μg STAT6 expression vector. **E **denotes the Emr1 minimal promoter and **T1-11 **denote mTARC putative STAT6 sites within the constructs. **F **refers to the forward orientation of the promoter. Data are representative of at least 2 transfections and results are displayed as fold induction of sample relative luciferase activity (luciferase/β-galactosidase) with cytokine treatment +/- SD.

Oligonucleotides consisting of three copies of the EMSA sequence for each of the sites were cloned onto a heterologous promoter, the minimal Emr1 -40 promoter [58], in pGL3 Basic. The Emr1 -40 minimal promoter was selected for its low basal activity in Mθ, lack of inducibility by IL-4 (Figure 8A) and absence of TATA box (similar to mTARC). Constructs containing sites in forward and reverse orientations were identified. When co-transfected with STAT6 into RAW 264.7 cells (Figure 6), trimers of T1 or T6 in either the forward (3XT1 F and 3XT6 F) or reverse orientations (data not shown) were not induced by IL-4 at all. In contrast, trimers of T2, T4 and T11 were induced by IL-4 regardless of insert orientation (data shown for forwards orientation only, 3XT2 F, 3XT4 F and 3XT11 F). The T4 trimer was only minimally induced by IL-4 (~3.5-fold), whereas T2 and T11 trimers were more highly induced than the mTARC -1173 full-length promoter construct. Thus, sites equally capable of binding STAT6 *in vitro *can have quite disparate capacity for activation by IL-4 in Mθ when assayed by transfection.

**Figure 7:**
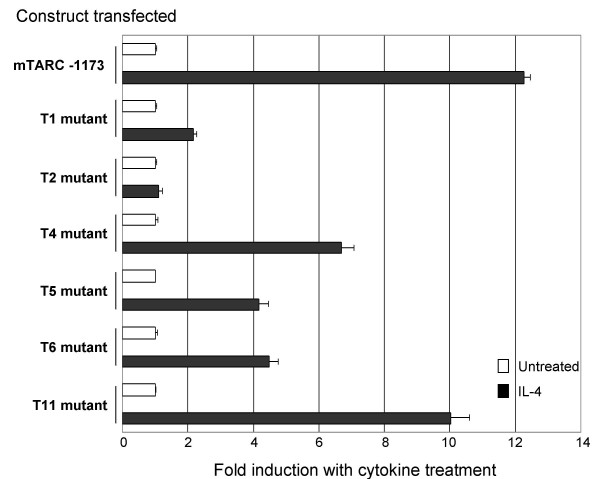
**Site-directed mutagenesis reveals a critical role for T1 and T2 and significant contributions from T4, T5 and T6 sites in the IL-4 induction of mTARC -1173**. RAW 264.7 cells were transfected as described in Figure 4A with 5 μg luciferase-conjugated mutant constructs **(T1-11 mutant) **and 3 μg STAT6 expression vector. Data are representative of at least 2 transfections and results are displayed as fold induction of sample relative luciferase activity (luciferase/β-galactosidase) with cytokine treatment +/- SD.

To resolve the contribution of individual putative STAT6 sites to overall mTARC promoter IL-4 inducibility, we performed site-directed mutagenesis of key sites within the context of the full-length -1173 promoter. The T1, T2, T4, T5, T6 and T11 sites were mutated, as these sites were implicated as strong candidate functional STAT6 by previous experiments (see above). As the 5' TTC nucleotide triplet is highly conserved amongst both published STAT6 sites [40,59,60] and all mTARC putative sites capable of binding STAT6 in EMSA experiments, this sequence was mutated to 5' AAC in order to abrogate STAT6 binding [61]. Mutant and wild-type sequence information was compared using the Genomatix MatInspector [62] to confirm that mutagenesis did not create new transcription factor binding sites that could alter the IL-4 responsiveness of the constructs (data not shown). Each mutant construct was co-transfected with STAT6 into RAW 264.7 cells and stimulated with IL-4 for 18 hours (Figure 7).

This assay confirmed that T1 and T2 are of critical importance to the IL-4 induction of mTARC -1173. Mutation of T2 resulted in complete abrogation of promoter activation by IL-4, whereas T1 mutation reduced promoter fold induction by IL-4 from 12-fold (mTARC -1173 wild-type) to 2-fold. This is consistent with results shown in Figures 4 and 5, but in contrast with the functional activity of the isolated T1 trimer in Figure 6. Mutation of T4 reduced promoter IL-4 induction by 40%, indicating, as suggested by Figures 4 and 6, that T4 contributes less significantly than T1 and T2 to the overall IL-4 induction. Similarly, although T11 bound STAT6 in EMSA and was potently activated by IL-4 as an isolated trimer, it does not appear to have significant activity within the context of mTARC -1173 (Figures 4 and 6). Interestingly, mutation of either T5 or T6 significantly reduced mTARC -1173 promoter IL-4 induction from 12-fold to 4-fold, demonstrating that these sites do contribute to overall IL-4 induction (consistent with Figure 4), even though they cannot bind or be activated by STAT6 when isolated from the promoter. Thus, the functional activity of STAT6 sites in isolation may be distinct from their functional activity within the context of their endogenous promoter. A summary of this data is compiled in Table 1.

### Murine TARC STAT6 sites in their natural configuration constitute potent IL-4 response elements

**Figure 8:**
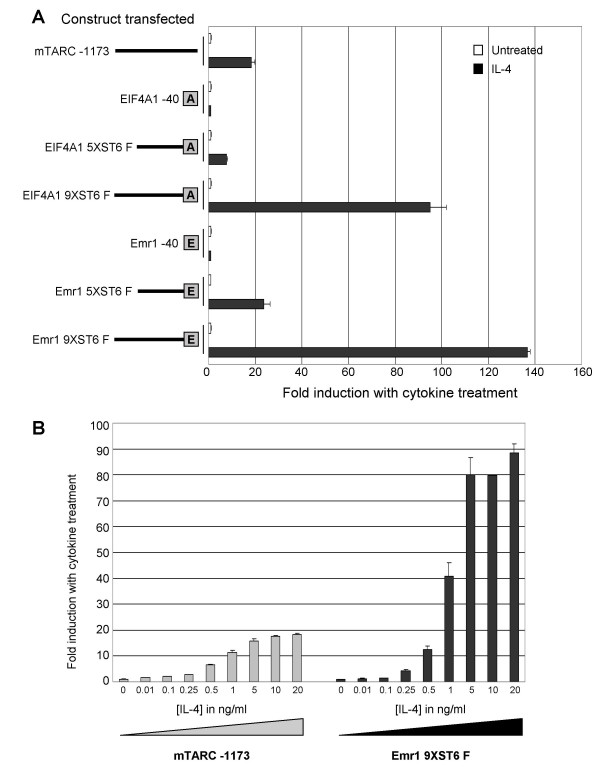
**mTARC STAT6 sites on heterologous minimal promoters are potent IL-4 responders**. RAW 264.7 cells were transfected as described in Figure 4A with 5 μg promoter-luciferase constructs indicated and 3 μg STAT6 expression vector. **E **denotes the Emr1 minimal promoter and **A **denotes the EIF-4A1 minimal promoter. The 5XST6 constructs consist of 394 bp murine TARC sequence from position -132 (refer to Figure 3) to -526 and contain 5 of the putative STAT6 sites. The 9XST6 constructs consist of 736 bp murine TARC sequence from position -132 (refer to Figure 3) to -868 and contain 9 of the putative STAT6 sites. **F **refers to the forward orientation of the promoter. Cells were treated +/- 20 ng/ml recombinant murine IL-4 (*A*) or IL-4 doses stated (*B*) for 18 hours before being harvested and assayed for luciferase and β-galactosidase activity. Data are representative of at least 2 transfections (*A*) or standardised results from multiple transfections (*B*) and results are displayed as fold induction of sample relative luciferase activity (luciferase/β-galactosidase) with cytokine treatment +/- SD.

We next tested whether the natural configuration of sites within the mTARC promoter could confer IL-4 inducibility on heterologous promoters and hence act as a transferable IL-4 response element. The Emr1 -40 (non TATA box) and the EIF4A1 -40 (TATA box) [63] minimal promoters were selected as neither was induced by IL-4 nor had strong basal activity when transfected into RAW 264.7 cells (Figure 8A). To assess the significance of the distal (non-binding) putative STAT6 sites and potential cooperation between the T5 and T6 sites in enhancing mTARC induction by IL-4, promoter constructs covering two different regions of the mTARC proximal promoter were designed. One region from position -132 (refer to Figure 3) to -526 contained the five most proximal putative STAT6 sites and bisected the sequence containing T5 and T6. A second region from position -132 to -868 contained nine putative STAT6 sites and intact T5-T6 sequence. The T11 site was not included in these constructs because it had not appeared significant in the experiments shown in Figure 7. Constructs were synthesised containing either the five (5XST6) or the nine (9XST6) putative STAT6 sites on each heterologous minimal promoter in both forward and reverse orientations.

When co-transfected with STAT6 into RAW 264.7 and stimulated with IL-4 (Figure 8A), these mTARC promoter elements were able to confer potent IL-4 inducibility on both the Emr1 and the EIF-4A promoters. Interestingly, only sites inserted in the forward orientation were induced by IL-4 (reverse constructs not shown). The 9XST6 constructs gave 95-135-fold induction with IL-4, whereas the 5XST6 constructs gave only 8-25-fold induction with IL-4. This suggests that distal mTARC promoter sites do contribute to the overall IL-4 response, possibly via cooperative interactions [41,64-67].

To examine the potency of the most IL-4 inducible construct, Emr1 9XST6 F, we performed dose-response experiments. The full-length mTARC -1173 and the Emr1 9XST6 F construct were transfected into RAW 264.7 cells and treated with IL-4 concentrations ranging from 0.01–20 ng/ml. Both constructs gave characteristic sigmoidal response curves that began to plateau above 5 ng/ml IL-4 (Figure 8B). The Emr1 9XST6 F construct showed greater induction by IL-4 than mTARC -1173 at all concentrations used above 0.25 ng/ml, which appeared to be the lower limit of the response. Importantly, the Emr1 9XST6 F construct still demonstrated a 40-fold induction with concentrations of IL-4 as low as 1 ng/ml, demonstrating that mTARC promoter sequences constitute a very sensitive IL-4 response element.

## Discussion

In this paper, we have shown for the first time that expression of the CC chemokine mTARC/CCL17 is potently and rapidly induced by IL-4 in primary murine macrophages and that this induction does not require *de novo *protein synthesis. TARC expression by alternatively-activated Mθ therefore represents an early component of Th2 inflammatory responses, which is exaggerated by further TARC expression by DC [30,31] and Th2 T-cells [43]. As TARC may represent an important therapeutic target in chronic allergic pathologies [22-24], we have analysed the mechanisms underlying its IL-4 induction in Mθ in some detail. Our experiments have revealed that synergistic interactions between at least five STAT6 sites within 1 kb of the mTARC transcriptional start site contribute to IL-4 regulation.

By real-time PCR, we measured significant up-regulation of mTARC mRNA within 4 hours of IL-4 stimulation, suggesting that Mθ expression of TARC is likely to be of considerable importance in the swift establishment of Th2 responses *in vivo*. This IL-4 induction was found to be dependent on expression of STAT6, a transcription factor strongly implicated in chronic Th2 pathologies including asthma [32,68-70]. TARC expression correlates with disease severity in many asthma patients [17-19,71]; and is one of the few chemokines found to be truly STAT6-dependent [70], hence the data presented here substantiate the link between TARC and STAT6 in disease and may suggest means by which TARC expression could be modulated for therapeutic benefit. STAT-mediated transcription is tightly regulated by intracellular suppressors [72-76]. The IFN-γ-mediated inhibition of mTARC expression may result from up regulation of SOCS1 [77,78], which we found inhibited the IL-4 induction of mTARC -1173 in RAW 264.7 cells (data not shown). SOCS1 is also up regulated by IL-4 [79,80], and so our observations of mTARC superinduction by IL-4 in the presence of cycloheximide could be related to reduced expression of this factor.

### Induction of mTARC by IL-4 is mediated by multiple STAT6 sites within the proximal promoter

No *de novo *protein synthesis was required for the IL-4 induction of mTARC, indicating direct interaction of STAT6 with the mTARC promoter. This was confirmed by ChIP experiments revealing STAT6 bound to the endogenous mTARC promoter in IL-4-treated Mθ. Fulkerson et al. [70] identified mTARC (CCL17) as a CC chemokine induced early in the lung in an allergen challenge model of asthma and using STAT6 deficient mice showed that mTARC expression in this disease model was STAT6 dependent. During the preparation of this manuscript, Wirnsberger et al. [43] reported the involvement of two STAT6 sites (one corresponding to the T2 site in our study) in the IL-4-mediated regulation of TARC in human T lymphocytes, but did not investigate the additional highly conserved proximal T1 site that we identified. The authors identified these STAT6 sites by 'visual inspection' rather than systematic analysis using transcription factor search engines and so the data do not exclude the possibility of more distal functional STAT6 sites that may diverge from strict consensus sequence [81]. We identified eleven putative STAT6 sites within the mTARC proximal promoter and determined that five of these had functional activity within the context of the mTARC -1173 construct. Hence the regulation of mTARC by IL-4 is more complex than previous work suggests, involving multiple STAT6 sites. To our knowledge, such interaction between STAT6 sites has only previously been reported for the promoter of the SOCS1 gene [80].

Of the five functional STAT6 sites identified, the two sites conserved between human and mouse, T1 and T2, were found to be important mediators of the mTARC induction by IL-4, even though T1 is not considered a classical STAT6 binding site [43]. By site-directed mutagenesis, T2 was shown to be absolutely essential for IL-4 induction of the mTARC promoter and mutation of T1 in the context of the -1173 promoter resulted in an 85% reduction in promoter induction by IL-4. The significance of the T2 site was predicted from its perfect TTC(N)_4_GAA consensus STAT6-binding sequence and ability to bind and be activated by STAT6 as an isolated site. In contrast, the T1 site has TTC(N)_3_GAA spacing, previously associated with weak or inhibitory activity when occupied by STAT6 [33,82], and also could not be activated by IL-4 as an isolated site, even though it bound STAT6.

Three additional sites (T4, T5 and T6) were found to support the IL-4 induction of mTARC to a lesser extent than T1 and T2, even though both T4 and T6 are perfect consensus STAT6-binding sites. Interestingly, the T5 imperfect STAT6 site was found to have equivalent function to T6 within the context of the mTARC -1173 promoter and the significance of both sites did not correlate with their capacities for STAT6 binding in EMSA. Thus, the findings of this paper sound a cautionary note for prediction of STAT6 site function from sequence data alone and emphasise the importance of adopting multiple approaches for the analysis of STAT6-mediated gene transcription. Notably, STAT6 has also been shown to bind to an imperfect palindrome site in the delta-opioid receptor [81], suggesting that functional STAT6 sites in other genes are also not restricted to a simple TTC(N)_4_GAA consensus sequence and cannot be identified as such.

The involvement of five closely-apposed STAT6 sites in the IL-4 induction of mTARC suggests that overall promoter induction may result from interactions between sites. Cooperation between proximal STAT6 sites in the SOCS1 promoter has been described [80] and other STAT proteins have similarly been shown to interact to activate gene transcription [41,64-67]. This arrangement of functional sites may stabilise STAT6 binding to low-affinity or non-canonical sites, such as T5, enhancing the overall transcriptional response to IL-4. Obligate stabilising interactions between STAT6 dimers bound at the juxtaposed T5 and T6 sites may explain the lack of STAT6 binding to these sites in isolation, as well as the comparable reduction in promoter induction by IL-4 when either site was mutated.

From our analyses of the functional activity of mTARC STAT6 sites, we were able to generate transferable promoter elements that conferred potent IL-4 responsiveness on two heterologous promoters. Constructs containing five mTARC putative STAT6 sites and intervening promoter sequences in their endogenous arrangement (5XST6) were induced up to 95-fold by IL-4, whereas constructs containing nine sites (9XST6) were induced up to 135-fold. The significantly greater activation of the 9XST6 constructs containing both T5 and T6 sites than the 5XST6 constructs lacking T6 may have resulted from interactions between the T5 and T6 sites. Although the 5XST6 constructs contained the T1, T2, T4 and T5 functionally significant STAT6 sites, stimulation with IL-4 resulted in promoter activity that was only 10–20% that of the 9XST6 constructs. Thus, distal promoter regions play an important role in mTARC induction by IL-4. Importantly, the potent IL-4 inducibility of these heterologous promoter constructs suggests that mTARC-derived IL-4 responsive elements could be used to direct IL-4-inducible gene expression *in vivo *with potential scientific and therapeutic benefit.

## Conclusion

In summary, we have demonstrated that Th2 cytokines regulate murine TARC by a direct-acting STAT6 pathway. We have shown that the mTARC promoter contains multiple functional STAT6 sites with heterogeneous capacity for binding of STAT6 and transcriptional activation. The sites conserved between human and mouse TARC promoters are the most crucial for the IL-4 induction of mTARC, but distal sites also play a significant role. Importantly, sites with imperfect consensus sequence have been shown to be functional, suggesting that the analysis of other STAT6-regulated genes may be warranted. These findings have also allowed us to develop highly responsive IL-4-inducible elements, significantly more active than those generated from multimerised individual STAT6 sites [33,83], which could be exploited direct transgene expression in alternatively activated macrophages *in vivo*.

## Methods

### Cells

All experiments were carried out according to Home Office guidelines and with appropriate local ethical approval. Male C57BL/6, BALB/c or STAT6^-/- ^[51] mice (8–12 weeks) were injected i.p. with 1 ml BIOgel beads (Bio-Rad) in PBS 2% w/v or thioglycollate broth (Difco) and elicited peritoneal macrophages (Mθ) were harvested by lavage with PBS/5 mM EDTA on day 4. Cells were usually plated at 2 × 10^6 ^Mθ per 35 mm dish in OptiMEM (Invitrogen) (determined by MTT^1 ^assay [84,85] to promote optimal cell viability over 72 hours) supplemented with L-glutamine and penicillin/streptomycin. Cells were washed 2 hours after plating and used in assays after 24 hours at 37°C. Cells were treated with recombinant cytokines (R&D Systems) or LPS (*S. typhimurium*, SigmaAldrich) at the concentrations and time periods stated. RAW 264.7 cells were cultured in RPMI (Invitrogen) supplemented with L-glutamine, penicillin/streptomycin, and 10% foetal calf serum. Cells were plated at 2 × 10^6 ^cells per 35 mm dish and left at 37°C for 30 minutes to adhere before cytokine treatment.

### RT-PCR and real-time PCR

Total RNA was prepared by scraping cells into RNAzol (Biogenesis) according to manufacturer's instructions. RNA was DNAse-treated (Promega) and 1 μg was reverse transcribed using primer (dT)_15 _for cDNA synthesis (Roche) and M-MLV-RT enzyme (Promega). Resultant cDNA was used in 20 μl final volume semi-quantitative PCR reactions using Red Hot DNA Polymerase enzyme and reaction mix (ABGene), supplemented with 1.5 mM MgC1_2_, 0.2 mM each dNTP and 0.3 μM forward and reverse primers. Primers for semi-quantitative PCR were as follows: **β**_**2**_**M**^**1**^**(β**_**2**_**-microglobulin)**: 5' TGACCGGCTTGTATGCTATC and 5' CAGT GTGAGCCAGGATATAG (222 bp) and **mTARC**: 5' ATGAGGTCACTTCA GATGCT and 5' AGGTCACGGCCTTGGGTT TT (284 bp) **mTARC**: 5' GCTGCCGTCATTTTCTGCCT and 5' GCTAAACGCTTTCATTAAAT. **IP-10**: 5' GCTGCCGTCATTTTCTGCCT and 5' GCTAAACGCTTTCATTAAAT. β_2_M was amplified from 1 μl (~20 ng) cDNA in a 24 cycle PCR program with cycling conditions as follows: 95°C for 30 seconds, 54°C for 30 seconds, 72°C for 30 seconds. Normalised template volumes were subjected to 31–35 PCR cycles using mTARC and IP-10 primers.

Real-time PCR was carried out with 2.5 μl (~50 ng) cDNA in multiplex reactions containing both mTARC and HPRT^1 ^(hypoxanthine guanine phosphoribosyl transferase) primers (at 0.3 μM each) and fluorogenic probes (at 0.2 μM each) in 25 μl final volumes of Universal MasterMix kit (PE Applied Biosystems). Primer and probe sequences were as follows: **HPRT**: 5' GACCGG TCCCGTCATG, probe ACCCGCAGTCCCAGCGTCGTG and 5' TCATAACCTGGTTCATC ATCGC and **mTARC**: 5' GGGATGCCATCGTGTTTCTG, probe CTGTCCAGGGCAAGCT CATCTGTGC and 5' GCCTTCTTCACATGTTTGTCTTTG. PCR and TaqMan analyses were performed using the ABI/PRISM 7700 sequence detector system (PE Applied Biosystems). Reactions were carried out in triplicate on 96-well plates with PCR cycling conditions: 50°C for 2 minutes, 95°C for 10 minutes, then 95°C for 15 seconds, 62°C for 1 minute for 50 cycles. Relative mTARC cDNA copy number was interpolated from a standard curve generated from cycle threshold values of serial dilutions of a known mTARC-containing cDNA sample and normalised to relative HPRT copy number.

### Enzyme-Linked Immunosorbent Assay (ELISA)

C57BL/6 BIOgel-elicited Mθ were cultured in 1 ml supplemented OptiMEM, which was removed at specified time points, centrifuged at 16000 *g *at 4°C for 1 minute to remove cell debris and then frozen in aliquots at -70°C. ELISA was carried out with 50 μl each supernatant sample using the mouse TARC/CCL17 Quantikine kit (R&D Systems). Samples were analysed in duplicate at 450 nm. Levels of mTARC protein in unknown samples were quantified by interpolation from a standard curve generated in the same experiment.

### Chromatin immunoprecipitation (ChIP)

Balb/c bone marrow Mθ were cultured for 7 days in L cell-conditioned medium. Cells were treated with 10 ng/ml recombinant murine IL-4 for the times indicated prior to formaldehyde fixation and sonication as described previously [86]. Immunoprecipitation was performed using the M20 anti-STAT6 antibody (Santa Cruz) followed by reversal of cross-linking and PCR amplification of the proximal TARC promoter using the **mTARC **primers 5' GAGGTGACCTATTTAACCTCTC and 5' GAAGTAAGTCATTGCCCTTACC An M20 STAT6 blocking peptide (Santa Cruz) was used to confirm the specificity of the immunoprecipitation.

### Translation inhibition experiments

C57BL/6 thioglycollate-elicited Mθ were treated with 0, 0.5, or 1 μg/ml cycloheximide (BDH) for 2 hours prior to addition or not of 20 ng/ml recombinant murine IL-4. Cells were incubated at 37°C for 4 or 24 hours before RNA was harvested and processed for real-time PCR.

### Identifying putative STAT6 binding sites in the murine TARC promoter

Mouse chromosome 8 sequence information was obtained from the Celera database and verified using the Sanger Centre database [52]. Murine sequence was aligned with the homologous human chemokine cluster on chromosome 16q13 to search for conserved sequence amongst the human and murine TARC promoters. Sequences were analysed for potential transcription factor binding sites using Genomatix MatInspector [62].

### Luciferase constructs and transient transfections

Murine TARC promoter fragments were PCR amplified from 200 ng C57BL/6 genomic DNA sample using a 1:1 ratio of Herculase Enhanced DNA Polymerase (Stratagene) and Taq Polymerase (Gibco BRL) in 25 μl reactions containing 1% DMSO, 0.2 μM each dNTP and 100 ng/ml each primer. The reverse primer for full-length (-24 to -1173) and 5' deletion series mTARC promoter constructs was: 5' AACTAAGCTTTCTTCATGGGTCCTTCTGCCT. Forward primer sequences were as follows: **-1173**: 5' AACTACGCGTTCTGCTAGCACATG AGTGCAA, **-839**: 5' AACTACGCGTTGGGTATGCTGAAAATTCACA, **-649**: 5' AACTAC GCGTTTCCAACCCACTAAAGCACA, **-498**: 5' AACTACGCGTGCCT CTTCTACTGAAC A, **-467**: 5' AACTACGCGTTCACCCTCCTGTGGACCTTCT and **-128**: 5' AACTACGCGTT GGAAGGATTATAGGAGGGGA. Purified PCR products were digested with *Mlu *I and *Hind *III and cloned into pGL3 Basic (Promega), which contains the firefly luciferase gene.

Site-directed mutagenesis was carried out by 2-stage PCR using mTARC -1173 forward and reverse cloning primers noted above and forward and reverse primers designed to introduce 2-base pair mutations within selected mTARC STAT6 sites. The mutant primers were as follows (base changes underlined): **T1**: 5' AGTTATCTCAAAAACTT TGAATTTCT and 5' AGAAATTCAAAGTTTTTGAGATAACT, **T2**: 5' GTCGAGTG ACCAAACTCTGGAAAGCC and 5' GGCTTTCCAGAGTTTGGTCACTCGAC, **T4**: 5' GGACTAGGCCTCAACTACTGAACA TC and 5' GATGTTCAGTAGTTGAG GCCTAGTCC, **T5**: 5' TGAACCTCTGGGAACCCCAAAATAGG and 5' CCTATTTT GGGGTTCCCAGAGGTTCA, **T6**: 5' GACAGGCTCAGCAACAGCTGAACCTC and 5' GAGGTTCAGCTGTTGCTGAGCCTGTC, **T11**: 5' GCCGGTTCTTTAACTCAGTA AACCC and 5' GGGTTTACTGAGTTAAAGAACCGGC. In the first stage, two PCR reactions were carried out to generate mutants of each site: mTARC -1173 forward with mutant reverse and mutant forward with mTARC -1173 reverse. The products of these reactions were used in a 1:1 ratio as template for the second-stage PCR reaction using only mTARC -1173 forward and reverse cloning primers. The final PCR products were purified and digested with *Hind *III and *Mlu *I and cloned into pGL3 Basic.

Primers and oligonucleotides for 9XST6 and 5XST6 derivative constructs were designed to create 5' and 3' *Mlu *I sites for bi-directional cloning onto Emr1 -40 [58] and EIF4A1 -40 [63] minimal promoters in the pGL3 Basic vector (Promega). The minimal promoters were modified by addition of an *Mlu *I linker sequence (5' CACGCGTGGT AC) for ease of cloning. The reverse primer sequence for the 9XST6 and 5XST6 PCR reactions was: 5' AACTACGCGTAGAGAAATTCAAAGAATTTGAGA and the forward primers were: **9XST6**: 5' AACTACGCGTCTCTTTTCTGGGCACTGTTGT and **5XST6**: 5' AACTACGCG TTGGGTTCCCCAAAATAGGGT.

Trimerised mTARC STAT6 site constructs were generated by random self-ligation of 5'-phosphorylated, PAGE-purified trimers of the T1, T2, T4, T6 and T11 site oligonucleotides used as probes in the EMSA experiments (see below for sequences). These trimers were designed to create 5' and 3' *Mlu *I sites when annealed to form double-stranded DNA for cloning onto the Emr1 -40 minimal promoter (as described above).

All plasmids were sequenced and prepared using Qiagen or QBIOgene endotoxin-free kits to ensure levels of endotoxin contamination were ≤ 0.1 EU/μg. Transient transfections were carried out by electroporation of RAW 264.7 as described previously [33]. Ten million RAW 264.7 cells were routinely co-transfected with 5 μg specific mTARC luciferase promoter construct DNA and 3 μg of a murine STAT6 expression vector (gift of Yoshihiro Ohmori [87]). In all experiments, 1 μg β-galactosidase expression vector was co-transfected as a control for transfection efficiency. Cells were incubated for 16–18 hours post-transfection +/- 20 ng/ml recombinant murine IL-4 before whole cell extracts were harvested in 250 μl reporter lysis buffer (Promega). Transfection results are shown as Relative Luciferase Activity (luciferase/β-galactosidase) or fold induction of this value with IL-4 treatment, as appropriate.

### Electrophoretic Mobility Shift Assays (EMSA)

C57BL/6 BIOgel-elicited Mθ were plated at l.5 × 10^7 ^cells per 90 mm dish and treated +/-20 ng/ml either recombinant murine IL-4 or recombinant murine IFN-γ for 1 hour. Cells were washed and incubated at 4°C in PBS supplemented with 5 mM EDTA and 0.2 mM PMSF for 15 minutes. Nuclear extracts were prepared by addition to each dish of 1 ml nuclear extract buffer I containing 10 mM HEPES pH 7.9, 1.5 mM MgCl_2_, 10 mM KC1, 0.5 mM DTT, 0.2 mM PMSF and additional protease inhibitors. Cells were incubated at 4°C for 15 minutes, then scraped into this buffer and vortexed gently. Cells were centrifuged at 2000 *g *for 30 seconds to recover the nuclei and the supernatant was discarded. Pellets were resuspended in 25 μl nuclear extract buffer II containing 20 mM HEPES pH 7.9, 25% glycerol, 420 nM NaCl, 0.5 mM DTT, 0.2 mM PMSF and protease inhibitors and left on ice for 20 minutes before being centrifuged at 16000 *g *for 15 minutes at 4°C. Supernatants from identical cytokine treatments were pooled and frozen at -70°C until used. Protein concentration of extracts was quantified using a BCA kit (Pierce) and 3 μg extract were used per EMSA assay.

Complementary forward and reverse oligonucleotides were designed for each mTARC putative STAT6 site. The forward sequences were as follows: **T1 probe**: 5' ATCTCAAATTCTTTGAATTTCTCTAG, **T2 probe**: 5' AGTGACCATTCTCTGGAAAGCCACAA, **T3 probe**: 5' TACTGAACATCCCTGTAACCTGCTCA, **T4 probe**: 5' TAGGCCTCTTCTACTGAACATCCCT G, **T5 probe**: 5' CCTCTGGGTTCCCCAAAATAGGGTGG, **T6 probe**: 5' GGCTCAGCTTCAGCTGAACCTCTGGG, **T7 probe**: 5' GTGCTCTATTTCTGGGAGCTGAGCTT, **T8 probe**: 5' CTATGGGCTTCTAGGGCAGCCTCTGG, **T9 probe**: 5' AAACTCTTTTCTGGGCACTGTTGTTA, **T10 probe**: 5' ATAGTGACTTGTCCTGAAACTCTTTT and **T11 probe**: 5' GGTTCTTTTTCTCAGTAAACCCTGGC. Forward and reverse oligonucleotides were annealed and end-labelled with ^32^P (Amersham Pharmacia Biotech). Nuclear extracts were incubated with labelled oligonucleotides in sample buffer with final concentrations of 20 mM HEPES pH 7.9, 6.25% glycerol, 0.5 mM EDTA, 50 μg/ml poly dIdC, 50 μg/ml BSA and 12.5 μg/ml salmon sperm DNA for 15 minutes at room temperature. For supershift assays, 400 ng specific or control antibodies (Santa Cruz) were also added to the EMSA reactions. For cold competition assays, 100-fold molar excess amounts of unlabelled oligonucleotides were added to appropriate reactions. Samples were separated on a 7% polyacrylamide gel run at 250 V (20 mA) for 3 hours at 4°C and visualised after exposure to X-ray film.

## Abbreviations

Ab, antibody

β_2_M, β_2_-microglobulin

CCR4, CC chemokine receptor 4

CLA, cutaneous lymphocyte antigen

DC, dendritic cell

HPRT, hypoxanthine guanine phosphoribosyl transferase

Mθ, macrophage(s)

MTT, 3-(4,5-dimethylthiazol-2-yl)-2,5-diphenyltetrazolium bromide

mTARC, murine homologue of TARC

PBMC, peripheral blood mononuclear cell

PI3 kinase, phosphatidylinosito1-3 kinase

SD, standard deviation

SEM, standard error of the mean

STAT, signal transducer and activator of transcription

Th1, T helper cell type 1

Th2, T helper cell type 2

TARC, thymus and activation-regulated chemokine.

## Competing interests

The authors have no competing financial or other interests relating to the work.

## Authors' contributions

KL designed and executed ELISA, EMSA, RT-PCR and transfection experiments, analysed the data and drafted the first version of the manuscript. JSW and SH performed ChIP experiments. JL was responsible for bioinformatics analyses. CKG contributed to the design and interpretation of experiments. DRG conceived of the study, was responsible for the design and coordination of the study and the submission and revision of the final manuscript. All authors read and approved the final manuscript.

## Supplementary Material

Additional File 1 Regulation of mTARC expression by IL-4 and IFN-γ. This figure shows the expression of mTARC and IP-10 mRNAs in total RNA prepared from peritoneal macrophages treated with the indicated amounts of recombinant murine IL-4 or IFN-γ. Click here for file

Additional File 2 *De novo *protein synthesis is not required for the IL-4 induction of mTARC. This figure shows mTARC mRNA expression in C57BL/6 peritoneal thioglycollate-elicited Mθ that were pre-treated with either 0, 0.5 or 1 μg/ml cycloheximide for 2 hours. Cells were then treated +/- 20 ng/ml recombinant murine IL-4 for a further 4 or 24 hours. Total RNA was harvested and TARC cDNA copy number was measured by real- time PCR +/- SEM. Results are representative of at least 3 similar experiments. Click here for file

## Figures and Tables

**Table 1:** Summary of results for mTARC putative STAT6 sites

**Putative site**	**Position within mTARC promoter**	*Sequence*	*Significance inferred from 5 'deletion series*	**Binds STAT6 in EMSA**	**Trimer construct induced by IL-4 in transfections**	**Reduction in IL-4 induction of mTARC -1173 when site mutated (approx.)**
**T1**	-140 to -148	**TTC**TTTGAA*	***+***	**+**	**-**	**85%**
**T2**	-194 to -203	**TTC**TCTG**GAA**	***+***	**+**	**++**	**100%**
**T3**	-473 to -482	A**TC**CCTGT**AA**	***-***	**-**	**ND**	**ND**
**T4**	-484 to -493	**TTC**TACT**GAA**	***-***	**+**	**+**	**40%**
**T5**	-513 to -522	**TTC**CCCAA**AA**	***+***	**-**	**ND**	**60%**
**T6**	-531 to-540	**TTC**AGCT**GAA**	***+***	**-**	**-**	**60%**
**T7**	-657 to -666	**TT**TCTGG**GA**G	***-***	**-**	**ND**	**ND**
**T8**	-763 to -111	**TTC**TAGGGC**A**	***-***	**-**	**ND**	**ND**
**T9**	-854 to -863	**TTC**TGGGC**A**C	***-***	**-**	**ND**	**ND**
**T10**	-870 to -879	**TT**GTCCT**GAA**	***-***	**-**	**ND**	**ND**
**T11**	-904 to -913	**TTC**TCAGT**AA**	***-***	**+**	**++**	**15%**

*residues in bold conform to the core consensus STAT6 binding site.

+ indicates positive result.

- indicates negative result.

++ indicates highly activated by IL-4.

**ND **indicates experiment not done.
