# Supplementary figures and images for: Interleukin-4 induction of the CC chemokine TARC (CCL17) in murine macrophages is mediated by multiple STAT6 sites in the TARC gene promoter
Source: BMC Mol Biol. 2006 Nov 29;7:45. doi: 10.1186/1471-2199-7-45 (PMC1698493; doi:10.1186/1471-2199-7-45)

## Slide 1
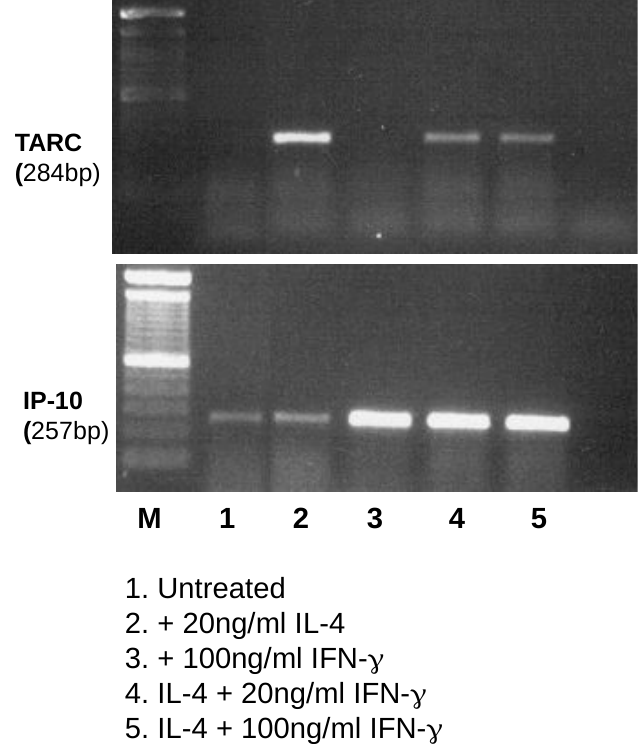

TARC
(284bp)
IP-10
(257bp)
 M 1 2 3 4 5
1. Untreated
2. + 20ng/ml IL-4
3. + 100ng/ml IFN-γ
4. IL-4 + 20ng/ml IFN-γ
5. IL-4 + 100ng/ml IFN-γ

Supplement: Additional File 1 — Regulation of mTARC expression by IL-4 and IFN-γ. This figure shows the expression of mTARC and IP-10 mRNAs in total RNA prepared from peritoneal macrophages treated with the indicated amounts of recombinant murine IL-4 or IFN-γ. [file 1471-2199-7-45-S1.ppt]

## Slide 1
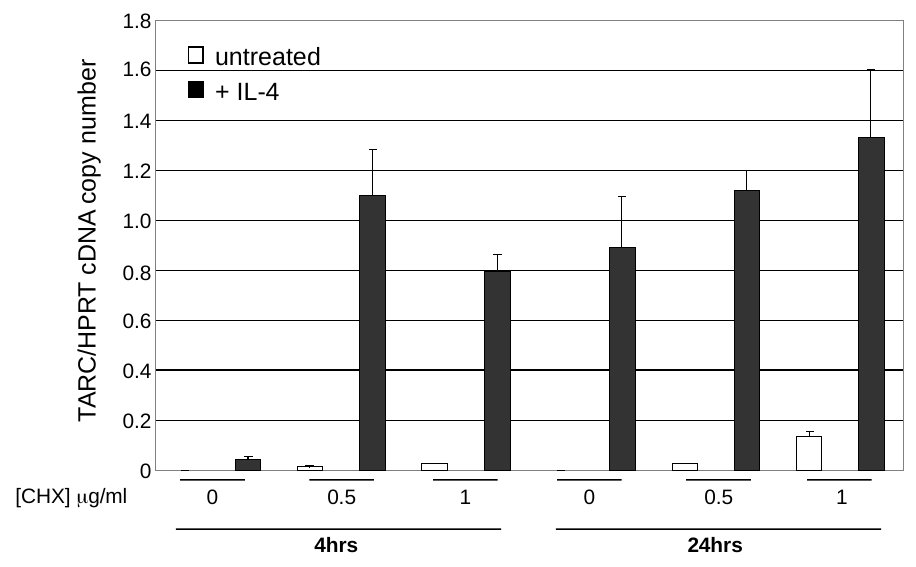

1.8
untreated
1.6
+ IL-4
1.4
1.2
1.0
TARC/HPRT cDNA copy number
0.8
0.6
0.4
0.2
0
[CHX] μg/ml
0
0.5
1
0
0.5
1
4hrs
24hrs

Supplement: Additional File 2 — De novo protein synthesis is not required for the IL-4 induction of mTARC. This figure shows mTARC mRNA expression in C57BL/6 peritoneal thioglycollate-elicited Mθ that were pre-treated with either 0, 0.5 or 1 μg/ml cycloheximide for 2 hours. Cells were then treated +/- 20 ng/ml recombinant murine IL-4 for a further 4 or 24 hours. Total RNA was harvested and TARC cDNA copy number was measured by real- time PCR +/- SEM. Results are representative of at least 3 similar experiments. [file 1471-2199-7-45-S2.ppt]
